# R-loops modulate *Trypanosome* antigenic variation

**DOI:** 10.1371/journal.pgen.1007809

**Published:** 2018-12-13

**Authors:** Lauren L. Prister, H. Steven Seifert

**Affiliations:** Department of Microbiology-Immunology, Northwestern University Feinberg School of Medicine, Chicago, Illinois, United States of America; University of Washington School of Medicine, UNITED STATES

Diversity generation systems employed by microbes are usually termed antigenic variation systems, and while there are many mechanisms used to promote the expression of various different forms of a gene product, many organisms have co-opted DNA recombination and repair factors to mediate specialized gene conversion reactions [1]. *Trypanosoma brucei* is the causative agent of African sleeping sickness and has one of the most well-studied antigenic variation systems that can produce thousands of versions of the variable surface glycoprotein (VSG) for immune avoidance. The VSG forms a coat on the outside of the parasite and since expression of VSG is essential [2], antigenic variation is thought to be important for long-term colonization in the host in the face of immune recognition. There are many questions that remain to understand the molecular aspects of the process. Briggs and colleagues show for the first time that RNA–DNA hybrids are involved in this complex gene diversification process [4].

**Fig 1:**
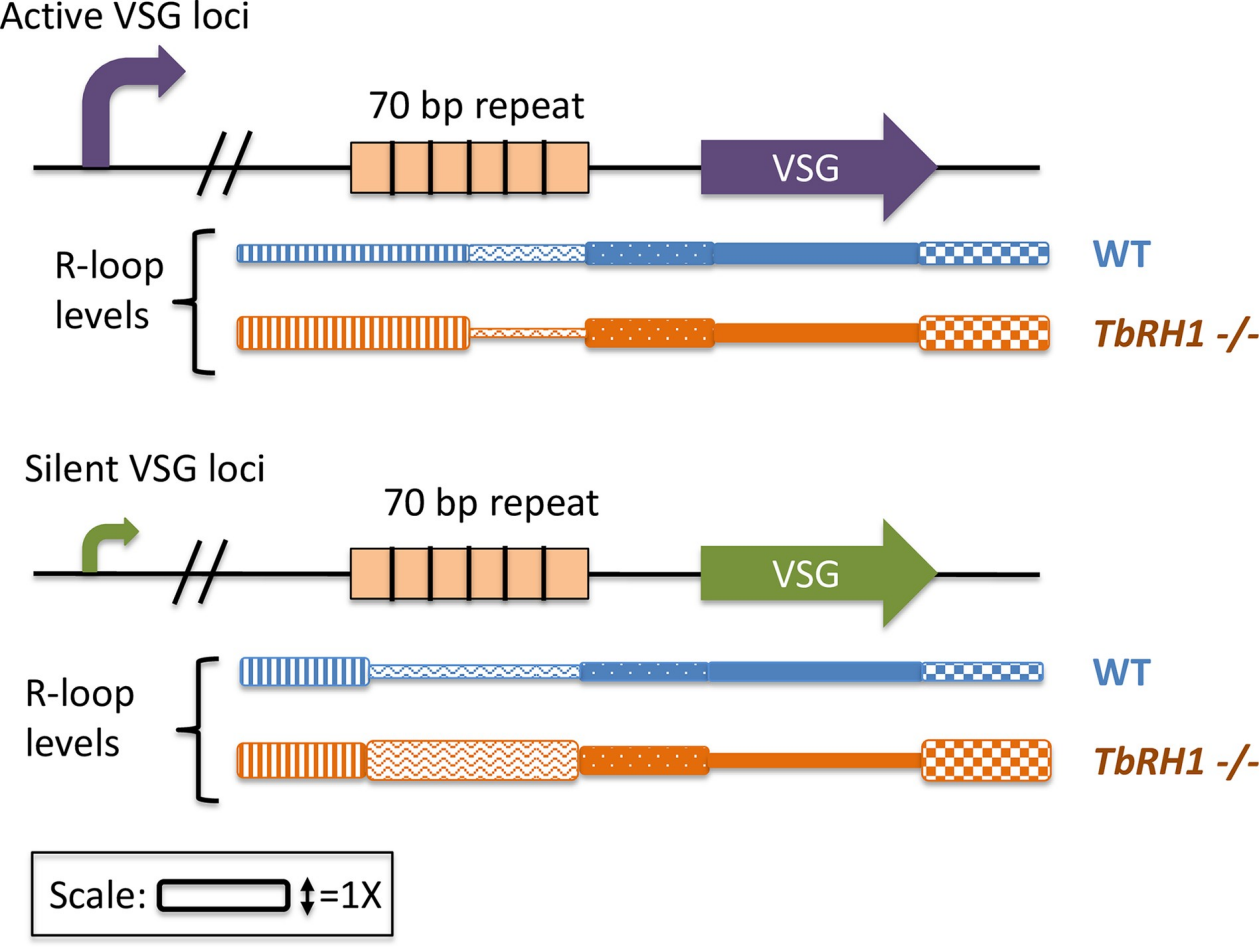
R-loop levels at silent and active ES. Briggs and colleagues performed DRIP-Seq and mapped the results to the active ES, VSG221 (Purple), and all silent ES loci (green). There are also expression site–associated genes located in the ES but are not pictured. R-loop levels were detected in WT cells and *TbRH1 −/−* cells (blue and orange, respectively). Below the loci, the relative R-loop levels for each region are shown; taller boxes represent increased detection of R-loops. 5′ to the 70-bp repeats R-loops were increased in the TbRH1 mutant (vertical stripes). When TbRH1 is absent, R-loops were increased over the 70-bp repeats in both the active and silent repeats (orange waves and stripes). 3′ of the VSGs in both silent and active loci, R-loops were increased, which are suggested to represent telomeric R-loops (orange checkered box). DRIP-Seq, DNA:RNA hybrid immunoprecipitation; ES, expressed copy; TbRH1, RNaseH1 enzyme of *T*. *brucei*; VSG, variable surface glycoprotein; WT, wild-type.

There are two major mechanisms for VSG antigenic variation: recombination of variant VSG sequences from subtelomeric storage copies into the telomeric expressed copy (ES) or a nonexpressed telomeric VSG and/or the activation of transcription of a different telomeric expression loci, with inactivation of the original ES [5]. Both expressed and silent telomeric expression sites have a similar genomic content and both include expression site–associated genes upstream of the VSG, a 70-bp repeat region, a single VSG gene, and the telomere (Fig 1) [6]. One locus is expressed at a time; however, multiple intact loci exist, and expression can change when RNA Polymerase I starts transcribing a different loci at a high level [7]. Though uncommon, coexpression of different VSGs from different promoters has been detected [8, 9].

VSG variation can also occur through recombination with other VSG genes, either from other telomeric ES loci or subtelomeric silent loci [10, 11]. Experimentally, an induced double-strand break can allow for trypanosome antigenic variation, but whether double strand breaks occur normally during antigenic variation has not been determined [3]. Recombination can replace the entire VSG gene or just portions of the gene, creating a mosaic VSG from multiple loci [10]. Some homologous recombination events utilize the recombination protein (Rad51) and the breast cancer susceptibility protein 2 (BRCA2); however, without these proteins, low levels of antigenic variation can occur, implicating a second unknown pathway [12, 13]. It is not clear if all VSG antigenic variation occurs by the same mechanism or whether the different reactions require different factors.

The report from Briggs and colleagues provides support for a role of RNA–DNA hybrids (R-loops) in *T*. *brucei* VSG antigenic variation. R-loops form when a guanine-rich (G-rich) RNA from a transcript remains bound to the DNA template [14]. R-loops are found in all levels of life and have been suggested to mediate a variety of molecular processes [15] but can also cause genome instability due to the stalling of replication forks or collisions of transcription and replication complexes [16]. For example, if mutations are made that increase R-loops (e.g., BRCA1/2 or senetaxin helicase [SETX]), a variety of human malignancies can result [17–21]. Repeat expansions, such as those underlying amyotrophic lateral sclerosis, can also result from R-loop accumulation [22]. A major role of the RNaseH family of exonucleases is to degrade the RNA of R-loops to prevent genomic instability [23]. R-loops have been proposed to act in the programmed recombination reactions of immunoglobulin (Ig)-heavy chain class switching [24, 25] and during *Neisseria* pilin antigenic variation [26].

Briggs and colleagues report that genetic inactivation of the RNaseH1 enzyme of *T*. *brucei* (TbRH1) enhances VSG antigenic variation, suggesting a plausible role for R-loops in modulating VSG variations. They used a monoclonal antibody to immunoprecipitate all RNA–DNA hybrids from a TbRH1 mutant cell (*TbRH1−/−*) or the parental cell, followed by deep sequencing to identify all R-loops throughout the genome (DRIP-Seq) [27]. They focus their report on the telomeric VSG-associated R-loops. They detect low levels of transcripts in TbRH1 wild-type cells but an enrichment of R-loops near both the expressed VSG gene and the silent telomeric VSG genes using DRIP-Seq and quantitative PCR (Fig 1). These results show that the silent telomeric VSGs are transcribed, that these G-rich RNAs form R-loops near both the expressed and silent telomeric VSGs, and that R-loops are efficiently removed by RNaseH1. A more detailed analysis showed that there was similar enrichment of R-loops within the 70-bp repeats at both silent and the expressed telomeric VSG but also detected increased R-loop formation in the actively transcribed expressed VSG relative to the nonexpressed VSGs.

Importantly, the *TbRH1−/−* mutant showed increased VSG antigenic variation, both by promoter switching and recombination, strongly suggesting that some of the R-loops detected by DRIP-Seq may be responsible for promoting antigenic variation. However, it was not possible in this work to differentiate cause from effect. In the future, overexpression of TbRH1 could be used to determine if R-loops are indeed necessary for antigenic variation. To support the hypothesis that R-loops direct antigenic variation, they analyzed levels of the DNA repair–associated histone, γ-H2A in VSG loci by chromatin immunoprecipitation and sequencing (CHIP-Seq) and immunofluorescence. In TbRH1 *−*/*−* trypanosomes, this DNA repair-associated histone was mainly localized to the actively expressed ES. Taken together, the results from this work led the authors to propose two models to explain the role of R-loops in antigenic variation. The first model postulates that R-loops in the active ES promote damage and that this initiates recombination from silent VSG copies. The second model proposes that R-loops trigger the transcriptional activation of silent telomeric VSGs through a shared direct mechanism, involving DNA damage or an unknown, indirect mechanism. Since Briggs and colleagues have not directly shown what sort of damage occurs (e.g., base changes, replication stalls, or interrupted repair processes), they have not directly linked the increased γ-H2A levels to the antigenic variation process.

Many questions remain to be answered for this antigenic variation system that is a paradigm for eukaryotic parasites. Do R-loops cause DNA lesions or are they formed as a result of the lesion? Instances of both have been documented in other systems [28, 29]. How might transcription or R-loops promote promoter switching? What occurs to the DNA after transcription and R-loop formation occurs to allow recombination or promoter switching? In a normal *TbRH1*-expressing cell, how does the cell handle R-loop formation and the competing production of mRNA to produce the essential VSG gene product? How is the donor sequence selected, and how does recombination occur? These and other questions will be answered in future studies, but this report makes a strong case for R-loops having an important role in VSG antigenic variation.
